# Evidence-Informed Deliberative Processes for Legitimate Health Benefit Package Design − Part I: Conceptual Framework

**DOI:** 10.34172/ijhpm.2021.158

**Published:** 2021-11-10

**Authors:** Rob Baltussen, Maarten Jansen, Wija Oortwijn

**Affiliations:** Department for Health Evidence, Radboud Institute for Health Sciences, Radboud University Medical Center, Nijmegen, The Netherlands.

**Keywords:** Evidence-Informed Deliberative Processes, Health Benefit Package, Health Technology Assessment, Universal Health Coverage, Stakeholder Involvement, Legitimacy

## Abstract

**Background:** Countries around the world are increasingly rethinking the design of their health benefit packages to achieve universal health coverage (UHC). Health technology assessment (HTA) bodies support governments in these decisions, but employ value frameworks that do not sufficiently account for the intrinsically complex and value-laden political reality of benefit package design.

**Methods:** Several years ago, evidence-informed deliberative processes (EDPs) were developed to address this issue. An EDP is a practical and stepwise approach for HTA bodies to enhance legitimate health benefit package design based on deliberation between stakeholders to identify, reflect and learn about the meaning and importance of values, and to interpret available evidence on these values. We further developed the conceptual framework and initial 2019 guidance based on academic knowledge exchange, analysing practices of HTA bodies, surveying HTA bodies and experts around the globe, and implementation of EDPs in several countries around the world.

**Results:** EDPs stem from the general concept of legitimacy, which is translated into four elements – stakeholder involvement ideally operationalised through stakeholder participation with deliberation; evidence-informed evaluation; transparency; and appeal. The 2021 practical guidance distinguishes six practical steps of a HTA process and provides recommendations on how these elements can be implemented in each of these steps.

**Conclusion:** There is an increased attention for legitimacy, deliberative processes for HTA and health benefit package design, but the development of theories and methods for such processes remain behind. The added value of EDPs lies in the operationalisation of the general concept of legitimacy into practical guidance for HTA bodies.

## Background

**Key Messages**

** Implications for policy makers**

- Evidence-informed deliberative processes (EDPs) provide a practical stepwise approach for health technology assessment (HTA) bodies to improve legitimacy of their decision-making processes.
- HTA bodies can improve this legitimacy through implementation of four elements in its processes: stakeholder involvement; evidence-informed evaluation; transparency; and appeal.
- Stakeholder involvement is the core element of EDPs and is ideally organised through stakeholder participation with deliberation. Alternative approaches are stakeholder consultation and stakeholder communication.

** Implications for the public**

Countries need to make important decisions as to which health technologies are included in the health benefit package. This often takes place without involvement of relevant stakeholders, eg, specific population groups who bear the consequences of decisions such as patients, the public and health professionals. As a consequence, important stakeholders values and knowledge may be ignored, and resulting decisions may not reflect societal preferences. Evidence-informed deliberative processes (EDPs) are developed to support counties to address this issue by improving the legitimacy, or fairness, of its decision-making process. Core element is the organisation of stakeholder participation in which eg, patients, health professional and the public deliberate and interact with policy-makers on benefit package design.

Countries around the world are increasingly rethinking the design of their health benefit packages to support the progressive realisation of universal health coverage (UHC), ie, to select the appropriate set of services at fair levels of coverage and financial protection.^1-3^ Many countries have established health technology assessment (HTA) bodies to support governments in these choices.^4^ HTA assesses the value of a health technology and can inform decisions at different levels, eg, reimbursement decisions on a single health technology or regarding larger parts of the benefit package.

 Decision-making on health technologies is an intrinsically complex and value-laden political process that takes place in an environment of diverging social values and interests.^5-9^ However, value frameworks currently employed by HTA bodies do not sufficiently account for this complex reality. They are typically based on the use of ‘substantive’ criteria, which are believed to reflect the most important social values. This has led HTA bodies to use, for example, ‘clinical benefit,’ ‘safety’ and ‘cost-effectiveness’ as important decision criteria.^4^ These frameworks are ill fitted for considering the broad diversity of stakeholder values and may lead to insufficient sets of relevant information and sub-optimal decisions.^5-7,10,11^

 Several years ago, in response to these shortcomings, evidence-informed deliberative processes (EDPs) were developed.^12^ An EDP is a practical and stepwise approach for HTA bodies to enhance legitimate health benefit package design based on deliberation between stakeholders to identify, reflect and learn about the meaning and importance of values, and to interpret available evidence on these values. Although the use of EDPs is relatively new, deliberative methods have been developed and used to some extent in the field of HTA since the 2000s.^13^ This paper reports on the further development of EDPs with an emphasis on the key concept of stakeholder participation with deliberation. This is based on academic knowledge exchange,^14-30^ analysing practices of eight HTA bodies,^31^ surveying a total of 27 HTA bodies and 66 experts around the globe,^21,32^ and experience with country level implementation of EDPs. With regard to the latter, EDPs are presently employed by national health authorities in Ghana, Iran, Moldova, Pakistan and Ukraine for revision of their health benefit packages,^33^ and its principles were previously applied for similar use in Kazakhstan, Thailand,^34^ the Netherlands^35^ and Indonesia.^23^

 A companion paper reports on the development of a practical guide to support HTA bodies with the implementation of EDPs, including best practice examples from countries around the world – released at the HTAi meeting in June 2021.^31^ Both papers target researchers, analysts and policy-makers involved in benefit package design at Ministries of Health and/or related HTA bodies, and can together be read as a ‘How to’ guide for organising legitimate decision-making processes.

 The paper starts with describing the conceptual framework of EDPs, including its elements and steps. It then focuses on the core element of EDPs, ie, stakeholder involvement ideally operationalised through stakeholder participation with deliberation. We conclude with several remarks on the added value of EDPs.

###  Conceptual Framework of Evidence-Informed Deliberative Processes 

 Authorities take decisions in health benefit package design on behalf of the population they serve. They should provide stakeholders – specific population groups who bear the consequences of decisions – with well-justified, reasonable reasons to appreciate the decision-making process as fair and confer legitimacy to the process despite favouring a different decision-making outcome.^36,37^ If this is not taken seriously by decision-makers, they risk losing their moral authority for making decisions. Subsequently, they may undermine the legitimacy of their own decision-making process.

 Stakeholders are often categorised as one of the 7Ps.^38^ These are:

- Patients, the public and carer: patients are individuals with the lived experience of a disease or disorder who can provide information in the HTA process pertinent to that disease or disorder; public is an umbrella term which, in the context of the HTA process, incorporates all nonpatient, non-commercial, and nonprofessional stakeholders within the health sector; a carer or caregiver is usually recognized as holding the interests of the patient(s) in their care but may have additional needs and interests.^39^
- Providers/health professionals: can bring in new perspectives, such as expert views on the effectiveness of technologies and the feasibility of alternative implementation options, including organisational aspects.
- Purchasers: can provide information on the feasibility of alternative implementation options, including organisational aspects.
- Payers: can provide generic non-disease specific perspectives and are often the public.
- Policy-makers: can provide information on the feasibility of alternative implementation options.
- Product makers (industry): can provide in-depth knowledge on the intervention under evaluation.
- Principal investigators (academia): can provide technical expertise on a variety of topics.

These stakeholders often have diverging social values and interests that result in different perceptions of what makes health technologies valuable.^40^ In pluralistic societies, stakeholders can be expected to disagree about what values can be used to include or exclude technologies from the package.^8^ For example, in decisions on public funding of expensive cancer drugs, patients may argue that the best treatment should be made available, while other patients may argue that their treatment should not be displaced and taxpayers may reason that it is more important to use public resources efficiently. In comparison to all these other parties, health professionals may want to have access to the latest technological developments in their field. As a result, decisions are bound to be controversial because stakeholders likely disagree about what should be prioritised, who should benefit and who should not.

 The accountability for reasonableness (A4R) framework recognises that stakeholders often justifiably disagree about the importance of specific social values in setting priorities and it argues that stakeholders are more likely to accept priorities that are the outcome of fair, legitimate processes.^41^ In other words, stakeholders may agree with outcomes of a fair process even though they may have preferred another outcome. The A4R framework identifies four key conditions for organising fair processes: (*i*) all relevant values should be considered; (*ii*) transparency must be ensured; (*iii*) appeal opportunities must be organised; and (*iv*) these conditions must be regulated. However, there is little practical guidance as to how these conditions should be implemented.

**Figure 1:**
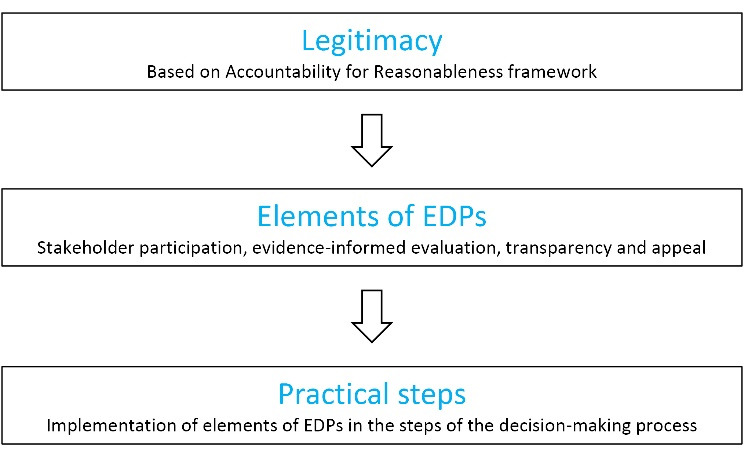
Conceptual Framework of Evidence-Informed Deliberative Processes. Abbreviation: EDPs, evidence-informed deliberative processes.

 EDPs respond to this and provide a practical stepwise approach for HTA bodies to implement the A4R conditions in their decision-making processes (Figure 1).^12^ As such, the use of EDPs, embodying fair processes, can improve the legitimacy of decision-making processes.

###  Elements of Evidence-Informed Deliberative Processes

 An EDP integrates four elements (Figure 1). First, the core element of EDPs is *stakeholder involvement *ideally operationalised through stakeholder participation with deliberation. Such stakeholder involvement ensures that all relevant values are considered, and is thereby a practical translation of the A4R relevance condition. By emphasizing the importance of stakeholder participation, EDPs go one step further than Daniels^41^ who recommends to consult those who are affected by a decision. We argue that participation is a much more powerful approach to stakeholder involvement than consultation – this is discussed in more detail below. Second, *evidence-informed evaluation *which allows for the use of scientific evidence and contributions from stakeholders in terms of their experiences and judgments when further evidence is unavailable. This ensures that relevant evidence is considered and is also a practical translation of the A4R relevance condition. Third, *transparency *whichensures that the deliberative processes, including their objectives, modes of stakeholder involvement, the decision reached and its related argumentation, is explicitly described and made publicly available. Fourth, *appeal* which ensures that a decision can be challenged and revised if new information or insights become available. These elements are direct operationalisations of the related A4R conditions. As such, EDPs provide the best way to combine evidence, information, perspectives and values, while also allowing these aspects to be identified and openly discussed.

###  Practical Steps

**Figure 2:**
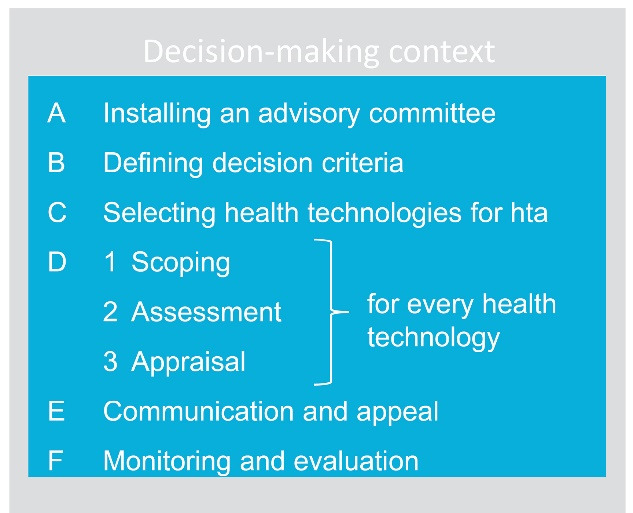
Six Steps of Implementing Evidence-Informed Deliberative Processes.

 We distinguish six practical steps of a HTA process based on existing HTA methods and tools, (Figure 2) and provide recommendations on how elements of EDPs can be implemented in each of these steps. The latter is based on observed practices of HTA bodies around the world. We speak of ‘HTA’ when referring to the whole process, while ‘hta’ specifically refers to the evaluation of a single technology. In steps A–C, we offer advice on the installation of an advisory committee, including the organisation of stakeholder involvement; on how to define decision criteria, and on how to set up a process for identifying and selecting health technologies for hta. In steps D1–D3, we give advice on how to scope, assess and appraise a specific health technology. In steps E–F, we provide guidance on communication and appeal, and monitoring and evaluation respectively. It is well recognised that, while the steps are presented as separate activities and in a linear fashion, in practice there may be iteration of them.^42^ The development and organisation of the EDP practical steps is described in detail in the companion paper.^31^

 Overall, the practical recommendations on the implementation of EDPs in each step of the HTA process should not be considered as a blueprint, but rather as inspirational. Each country has a unique decision-making context determined by factors such as local values and traditions, level of HTA development, and available knowledge, skills and resources. From this follows that each country should introduce its own appropriate form of EDPs in its process, eg, mode of stakeholder involvement and transparency.

###  Organising Stakeholder Involvement 

 Stakeholder involvement is formally defined as “an iterative process of actively soliciting the knowledge, experience, judgment and values of individuals selected to represent a broad range of direct interest in a particular issue, for the dual purposes of creating a shared understanding; and making relevant, transparent, and effective decisions.”^43^ It can improve the legitimacy of decision-making in three ways.^13^ First, stakeholder involvement can serve to identify the full range of interests that a society has in relation to a particular decision. Second, it can improve understanding among stakeholders by explicating the interests of all parties involved. Third, stakeholder involvement can contribute to improving the quality of decisions, as stakeholders contribute specific knowledge (eg, about barriers to implementation or meaningful patient outcomes).

 Generally, three approaches to stakeholder involvement are distinguished: participation, consultation and communication,^44^ and we discuss these below. Table provides examples on how HTA bodies can implement these approaches in the different steps of the HTA process.

**Table:** Options to Involve Stakeholders in Each Step of Evidence-Informed Deliberative Processes Based on Respectively Stakeholder Participation, Consultation and Communication^*^

	**Step A **	**Step B**	**Step C**	**Step D1 **	**Step D2**	**Step D3**	**Step E**	**Step F**
	**Installing an Advisory Committee**	**Defining Decision Criteria**	**Selecting Health Technologies for hta**	**Scoping**	**Assessment**	**Appraisal**	**Communication and Appeal **	**Monitoring and Evaluation**
**Participation**	Participation in advisory committee as *formal member* with voting rights. Typically for stakeholders who represent general interests							
	Participation in advisory committee as *non-formal* member without voting rights. Typically for stakeholders who represent interests of specific health technologies							
**Consultation**	Inviting stakeholders to nominate advisory committee members	Surveying stakeholders to assess their preferences vis-à-vis values and corresponding decision criteria	Inviting stakeholders to nominate health technologies	Interviews or focus groups, eg, using nominal group technique	Invited submissions	Providing written or oral statements to advisory committee	Inviting stakeholders to review draft lay summaries and communication materials	Inviting stakeholders to review draft impact pathways and derived indicators, definitions and operationalisations
				Invited submissions	Inviting stakeholders to review evidence reports	Expert panel consultation		
		Publishing tentative criteria and soliciting stakeholder input, eg, through citizen panels or patient advisory groups	Including stakeholder sources in horizon scanning	Multimedia analysis	Surveys	Citizen panels	Face-to-face meetings to discuss and address appeals and/or concerns, eg, public hearings	
					Social media analysis	Face-to-face meetings with stakeholders		
					Primary research/ synthesis	Inviting stakeholders to review draft recommendations		
**Communication**	Publishing a document describing the advisory committee installation process and its procedures	Publishing a document describing the definition process and the decision criteria	Publishing a document describing the selection process and selected health technologies	Publishing a document describing the scoping process	Publishing a document describing the assessment process and outcomes	Publishing a document describing the appraisal process and outcome	Publishing a document describing the communication and appeal process	Publishing a document describing the monitoring and evaluation process
							Multimedia dissemination of final decision reports	
							Publishing of appeals and relevant actions taken	

* Adapted from: Abelson et al.^44^

###  Stakeholder Participation 

 Stakeholder participation means that stakeholders are actively engaged in deliberations during each EDP step. A deliberative process is defined as “a series of coordinated activities allowing a group of people (or relevant stakeholders) to receive and exchange information, to critically examine an issue, and to come to an overall group judgement.”^45^ Through this interaction and practical reasoning, stakeholders may deepen their understanding of their own preferences and those of others affected by decisions. They may replace uninformed opinions by views that are more rational and better supported by arguments and evidence, improving the quality of the decisions. There is good evidence that participants learn from deliberative engagement, including considering information that is contrary to their opinions and can change their opinions in line with this new information.^46^ For these reasons, stakeholder participation can best realise the full benefits of stakeholder involvement and contribute to the legitimacy of decision-making. Accordingly, we strongly advise HTA bodies to consider implementing stakeholder participation in their decision-making process.

 Stakeholder participation on coverage decisions can be organised through an advisory committee. Advisory committees are typically related to an HTA body and have the mandate to formulate recommendations to decision-making bodies, such as the Ministry of Health. Stakeholder participation in the advisory committee can be organised in two complementary ways.

- HTA bodies may choose to include specific stakeholders as *formal members *of their advisory committee. Such stakeholders typically represent the general interest of patients and sometimes industry, and not a specific interest regarding certain health technologies. These members have voting power.
- HTA bodies can also organise stakeholder participation by inviting specific stakeholders to participate in their meetings. There stakeholders are *not formal members* of the advisory committee and are not granted voting power but can participate in deliberations. Such stakeholders typically represent interests or have specific expertise of the health technology being deliberated on. Nevertheless, this form of stakeholder participation does give some degree of influence and ownership of decisions to stakeholders.

There are several approaches to structuring the decision-making process to facilitate stakeholder participation and deliberation in committee meetings. We advise using a systematic approach that ensures all participants and members express their preferences and considerations, such as Nominal Group Technique. Practical guidance on Nominal Group Technique is available elsewhere.^47^ In addition, HTA bodies should take into account several general principles for stakeholder participation and deliberation.^48^ These include:

- Transparency: the deliberative processes – including the objectives, modes of stakeholder involvement, the decision reached and its related argumentation – should be explicitly described and made publicly available.
- Inclusivity: all relevant values pertaining to decisions on a health technology should be taken into account. This requires that relevant stakeholders are meaningfully involved in the decision-making process. This means that barriers to effective participation should be removed.
- Learning:stakeholders are ideally provided opportunities to participate and interact in deliberations as this likely improves the understanding of preferences, arguments and/or evidence, thereby improving the quality of the decision-making process.
- Impartiality: the deliberative process used for each decision and those involved in it should be free from undue influences, both internal (eg, from the agency supporting the HTA process) and external (eg, from stakeholders with vested interests).

The use of these principles in the organisation of stakeholder participation and deliberation is not without challenges. Ensuring transparency may be challenging in settings where there is little or no tradition of open decision-making. Ensuring inclusivity can be resource-intensive for HTA bodies who need to proactively identify affected stakeholders and involve them in decision-making processes and allocate adequately trained staff to the organisation of stakeholder participation. In addition, participating stakeholders may find it difficult to invest the time required to familiarise themselves with procedures of the HTA body. Ensuring learning takes place amongst stakeholders can be challenging for HTA bodies, as this requires multiple perspectives being effectively represented by participating stakeholders and that all perspectives are accounted for in the deliberations. Ensuring impartiality may also be challenging in contexts in institutions with less experience in managing conflicts of interest. Our guide provides practical guidance on how to deal with these challenges.^31^

###  Stakeholder Consultation

 In many countries, meaningful stakeholder participation and actual deliberation between stakeholders is non-existent or in its infancy. Although not ideal, HTA bodies often rely on non-deliberative ways to involve stakeholder perspectives in their HTA processes such as through consultation.

 Consultation refers to a structured process for collecting feedback from groups of stakeholders on specific decisions without providing opportunities for meaningful deliberation with the HTA body’s advisory committee. One example is the provision of oral or written patient testimonies to an advisory committee, as organised by the National Health Care Institute in the Netherlands, Canadian Agency for Drugs and Technologies in Health in Canada, the National Institute for Health and Care Excellence in the United Kingdom, and Pharmaceutical Benefits Advisory Committee in Australia.^49^ Other examples include the use of surveys, stakeholder meetings and solicited feedback from stakeholders on HTA draft reports, including proposed recommendations. This latter approach is followed by the National Committee for Health Technology Incorporation in Brazil, and the Institute for Quality and Efficiency in Health Care in Germany among others.^49^

 The benefit of consultation is the large number of respondents that can be reached, however, there are some drawbacks. First, the timing and topic for input is predefined, which limits the scope of comments that stakeholders can provide. Second, consultation offers no opportunities for deliberation among stakeholders and the HTA body’s advisory committee, which limits transparency and the facilitation of mutual learning. Third, the quantity of feedback can be significant, so time should be reserved for providing feedback. If the HTA body in question has limited capacity to do so, this can restrict the scope of public consultation and affect its ability to contribute to the overall legitimacy of the process.

###  Stakeholder Communication

 Stakeholder communication refers to efforts by HTA bodies to inform stakeholders of their activities and results by using communication platforms. Communication can be achieved through public meetings, by preparing plain-language versions of reports to increase accessibility, by dissemination to high priority groups using patients’ organisations or by using social media. Communication is characterised by a very low-level of stakeholder involvement. This one-way flow of information means there is little chance of stakeholders influencing the decision-making process. Note that stakeholder communication contributes to the requirement of transparency, the third element of legitimacy as defined in EDPs, and can thus be seen as a necessary activity of HTA bodies.

 As an illustration, Box 1 describes the organisation of stakeholder involvement in the development of the UHC benefit package in Pakistan.

**Box 1. Stakeholder Involvement in UHC Benefit Package Design in Pakistan**

The project team designed an advisory committee structure including TWGs on four disease areas, a NAC, and a Steering Committee (step A). In this way, more than 100 disease experts and stakeholders from national, province and district level participated through deliberation in the committees. Next, the Ministry of Health conducted a survey among TWG and NAC members to assess the importance of decision criteria for the prioritisation of health technologies, and these members also participated in a meeting where decision criteria were communicated (step B). In addition, the Ministry of Health, together with key stakeholders, reviewed a DCP3 model benefit package on the technologies that should be assessed for inclusion in the UHC benefit package in Pakistan (step C). Subsequently, the project team collected evidence on these technologies. The TWGs then classified technologies into categories of ‘high priority,’ ‘medium priority’ and ‘low priority’ using a deliberative process and majority voting as a closure mechanism. The meetings were led by a trained facilitator, and a rapporteur recorded the participants’ arguments and votes. Next, the NAC reviewed TWG argumentation and recommendations and further prioritized the list of ‘high priority’ services taking into ac-count fiscal space, coverage and co-payment levels, and complementary investments in the health system (step D). The NAC developed recommendations for preferred packages, and these were presented to the Steering Committee for their approval upon consultation with an international advisory group.

---------------

Abbreviations: DCP3, Disease Control Priorities 3; UHC, universal health coverage; EDPs, evidence-informed deliberative processes; TWG, technical working group; NAC, National Advisory Committee.

## Conclusion

 There is an increased attention for legitimacy, deliberative processes for HTA and health benefit package design, but the development of theories and methods for such processes remain behind.^10,26,50^ This is in stark contrast with eg, the field of economic analysis of health technologies where the theory of welfare economics and methods for cost-effectiveness analysis are well-developed.^26^ This indicates the importance of this paper and related initiatives such as the ‘Joint HTAi-ISPOR Task Force on Deliberative Processes for HTA.^51^

 Cuyler observes in his commentary on EDPs that the complexity and multifaceted nature of the topic makes it challenging to develop a “single unifying theory of deliberative processes.”^26^ He argues that the design of deliberative processes also requires imagination and descriptive evidence. This is indeed reflected in our work: the conceptual framework of EDPs stems from the general concept of legitimacy, the definition of four elements is a practical translation of the A4R framework,^41^ the definition of practical steps is based on existing HTA methods and tools, whereas related recommendations on best practices are inferred from observed practices of HTA bodies around the world.

 We consider EDPs as complementary to other concepts and methods to support HTA bodies on health benefit package design such as reported in the publication *What’s in, what’s out* by the Center for Global Development,^3^ the World Health Organization (WHO) *Making Fair Choices *report^52^ and its handbook *Strategizing national health in the 21st century.*^53^ Specific guidance on the use of HTA for benefit package design is provided in the *HTA Toolkit* by International Decision Support Initiative,^54^ and the *HTA roadmap* by Management Sciences for Health.^55^ All of these guides cover critical aspects of health benefit package design, including institutional set-up, required decision-making processes, the necessity of stakeholder involvement, collection of evidence and monitoring and evaluation aspects. The added value of EDPs lies in its explicit foundation in the concept of legitimacy and related elements (including stakeholder involvement ideally operationalised through stakeholder participation with deliberation), as well as in the provision of recommendations on how these elements can be implemented in each of the EDP steps.

 The next step is to develop practical guidance for HTA bodies, on the basis of the conceptual framework as presented in this paper. We have made first efforts by analysing practices of eight HTA bodies around the world in terms of the six steps of EDPs and related elements of legitimacy, as described in the companion paper.^31^

## Ethical issues

 Ethical approval was not necessary as no primary data was used.

## Competing interests

 Authors declare that they have no competing interests.

## Authors’ contributions

 All authors have contributed equally.

## References

[1] Chalkidou K, Glassman A, Marten R (2016). Priority-setting for achieving universal health coverage. Bull World Health Organ.

[2] Verguet S, Hailu A, Eregata GT, Memirie ST, Johansson KA, Norheim OF (2021). Toward universal health coverage in the post-COVID-19 era. Nat Med.

[3] Glassman A, Giedion U, Smith PC. What’s In, What’s Out: Designing Benefits for Universal Health Coverage. Washington, DC, United States: Brookings Institution Press, Center for Global Development; 2017.

[4] World Health Organization (WHO). Global Survey on Health Technology Assessment by National Authorities. WHO; 2015.

[5] Holm S (1998). The second phase of priority setting Goodbye to the simple solutions: the second phase of priority setting in health care. BMJ.

[6] Mitton C, Donaldson C (2004). Health care priority setting: principles, practice and challenges. Cost Eff Resour Alloc.

[7] Kapiriri L, Martin DK (2007). A strategy to improve priority setting in developing countries. Health Care Anal.

[8] Daniels N (2000). Accountability for reasonableness. BMJ.

[9] Abelson J, Giacomini M, Lehoux P, Gauvin FP (2007). Bringing ‘the public’ into health technology assessment and coverage policy decisions: from principles to practice. Health Policy.

[10] Daniels N, Porteny T, Urritia J (2015). Expanded HTA: enhancing fairness and legitimacy. Int J Health Policy Manag.

[11] Daniels N, van der Wilt GJ (2016). Health technology assessment, deliberative process, and ethically contested issues. Int J Technol Assess Health Care.

[12] Baltussen R, Jansen MP, Bijlmakers L (2017). Value assessment frameworks for HTA agencies: the organization of evidence-informed deliberative processes. Value Health.

[13] Baltussen R, Jansen M, Bijlmakers L (2018). Stakeholder participation on the path to universal health coverage: the use of evidence-informed deliberative processes. Trop Med Int Health.

[14] Baltussen R, Jansen MP, Bijlmakers L, Tromp N, Yamin AE, Norheim OF (2017). Progressive realisation of universal health coverage: what are the required processes and evidence?. BMJ Glob Health.

[15] Baltussen R, Mitton C, Danis M, Williams I, Gold M (2017). Global developments in priority setting in health. Int J Health Policy Manag.

[16] Jansen MP, Baltussen R, Mikkelsen E (2018). Evidence-informed deliberative processes - early dialogue, broad focus and relevance: a response to recent commentaries. Int J Health Policy Manag.

[17] Jansen MP, Helderman JK, Boer B, Baltussen R (2017). Fair processes for priority setting: putting theory into practice comment on “expanded HTA: enhancing fairness and legitimacy. ” Int J Health Policy Manag.

[18] Jansen MP, Baltussen R, Bærøe K (2018). Stakeholder participation for legitimate priority setting: a checklist. Int J Health Policy Manag.

[19] Jansen MP, Bijlmakers L, Baltussen R, Rouwette EA, Broekhuizen H (2019). A sustainable approach to universal health coverage. Lancet Glob Health.

[20] Kapiriri L, Baltussen R, Oortwijn W (2020). Implementing evidence-informed deliberative processes in health technology assessment: a low income country perspective. Int J Technol Assess Health Care.

[21] Oortwijn W, Jansen M, Baltussen R (2020). Use of evidence-informed deliberative processes by health technology assessment agencies around the globe. Int J Health Policy Manag.

[22] Seixas BV, Mitton C, Danis M, Williams I, Gold M, Baltussen R (2017). Should priority setting also be concerned about profound socio-economic transformations? a response to recent commentary. Int J Health Policy Manag.

[23] Tromp N, Prawiranegara R, Siregar A (2018). Translating international HIV treatment guidelines into local priorities in Indonesia. Trop Med Int Health.

[24] Chalkidou K, Li R, Culyer AJ, Glassman A, Hofman KJ, Teerawattananon Y (2017). Health technology assessment: global advocacy and local realities comment on “priority setting for universal health coverage: we need evidence-informed deliberative processes, not just more evidence on cost-effectiveness. ” Int J Health Policy Manag.

[25] Lauer JA, Rajan D, Bertram MY (2017). Priority setting for universal health coverage: we need to focus both on substance and on process comment on “priority setting for universal health coverage: we need evidence-informed deliberative processes, not just more evidence on cost-effectiveness. ” Int J Health Policy Manag.

[26] Culyer AJ (2020). Use of evidence-informed deliberative processes - learning by doing comment on “use of evidence-informed deliberative processes by health technology assessment agencies around the globe. ” Int J Health Policy Manag.

[27] Goetghebeur M, Cellier M (2021). Deliberative processes by health technology assessment agencies: a reflection on legitimacy, values and patient and public involvement comment on “use of evidence-informed deliberative processes by health technology assessment agencies around the globe. ” Int J Health Policy Manag.

[28] Gopinathan U, Ottersen T (2017). Evidence-informed deliberative processes for universal health coverage: broadening the scope comment on “priority setting for universal health coverage: we need evidence-informed deliberative processes, not just more evidence on cost-effectiveness. ” Int J Health Policy Manag.

[29] Hall W (2017). Don’t discount societal value in cost-effectiveness comment on “priority setting for universal health coverage: we need evidence-informed deliberative processes, not just more evidence on cost-effectiveness. ” Int J Health Policy Manag.

[30] Schlander M (2021). HTA agencies need evidence-informed deliberative processes comment on “use of evidence-informed deliberative processes by health technology assessment agencies around the globe. ” Int J Health Policy Manag.

[31] Oortwijn W, Jansen M, Baltussen R. Evidence-informed deliberative processes for health benefit package design – part II: A practical guide. Int J Health Policy Manag. 2021. 10.34172/ijhpm.2021.159. PMC9808268 34923809

[32] Oortwijn W, van Oosterhout S, Kapiriri L. Application of evidence-informed deliberative processes in health technology assessment in low- and middle-income countries. Int J Technol Assess Health Care. 2020:1-5. 10.1017/s0266462320000549. 32715993

[33] Radboud University Medical center: Global Health Priorities. Country applications of evidence-informed deliberative processes. https://www.radboudumc.nl/global-health-priorities. Accessed March 31, 2021.

[34] Youngkong S, Baltussen R, Tantivess S, Mohara A, Teerawattananon Y (2012). Multicriteria decision analysis for including health interventions in the universal health coverage benefit package in Thailand. Value Health.

[35] Zorginstituut Nederland. Pakketadvies in de praktijk: wikken en wegen voor een rechtvaardig pakket. Zorginstituut Nederland; 2017.

[36] Bærøe K, Baltussen R (2014). Legitimate healthcare limit setting in a real-world setting: integrating accountability for reasonableness and multi-criteria decision analysis. Public Health Ethics.

[37] Peter F. Political Legitimacy. http://plato.stanford.edu/entries/legitimacy/. Accessed March 28, 2021. Published 2010.

[38] Concannon TW, Meissner P, Grunbaum JA (2012). A new taxonomy for stakeholder engagement in patient-centered outcomes research. J Gen Intern Med.

[39] Street J, Stafinski T, Lopes E, Menon D (2020). Defining the role of the public in Health Technology Assessment (HTA) and HTA-informed decision-making processes. Int J Technol Assess Health Care.

[40] Grin J, van de Graaf H, Hoppe R. Technology Assessment Through Interaction. A Guide. The Hague, the Netherlands: Rathenau Institute; 1997.

[41] Daniels N (2000). Accountability for reasonableness. BMJ.

[42] Oortwijn W, Determann D, Schiffers K, Tan SS, van der Tuin J (2017). Towards integrated health technology assessment for improving decision making in selected countries. Value Health.

[43] Deverka PA, Lavallee DC, Desai PJ (2012). Stakeholder participation in comparative effectiveness research: defining a framework for effective engagement. J Comp Eff Res.

[44] Abelson J, Wagner F, DeJean D (2016). Public and patient involvement in health technology assessment: a framework for action. Int J Technol Assess Health Care.

[45] Gauvin FP. Factsheet What is a deliberative process? Publication No. 1193. The National Collaborating Centre for Healthy Public Policy; 2008. http://www.ncchpp.ca/docs/DeliberativeDoc1_EN_pdf.pdf.

[46] INVOLVE UK. The impact of deliberation on participants’ opinions. https://www.involve.org.uk/resources/knowledge-base/what-are-affects-deliberation/impact-deliberation-participants-opinions.

[47] Dunham, Randall. Nominal Group Technique: A User’s Guide. University of Wisconsin. https://www.sswm.info/sites/default/files/reference_attachments/DUNHAM%201998%20Nominal%20Group%20Technique%20-%20A%20Users%27%20Guide.pdf.

[48] Bond K, Stiffell R, Ollendorf DA. Principles for deliberative processes in health technology assessment. Int J Technol Assess Health Care. 2020:1-8. 10.1017/s0266462320000550. 32746954

[49] Oortwijn W, Jansen M, Baltussen R. Evidence-Informed Deliberative Process: A Practical Guide for HTA Bodies for Legitimate Benefit Package Design. Nijmegen: Radboud University Medical Center; 2021. https://www.radboudumc.nl/global-health-priorities.

[50] Baltussen R, Jansen MP, Mikkelsen E (2016). Priority setting for universal health coverage: we need evidence-informed deliberative processes, not just more evidence on cost-effectiveness. Int J Health Policy Manag.

[51] Health Technology Assessment International (HTAi). Joint HTAi-ISPOR Task Force on Deliberative Processes for HTA. https://htai.org/deliberative-processes-for-hta-joint-task-force/.

[52] World Health Organization (WHO). WHO Consultative Group on Equity and Universal Health Coverage. Making Fair Choices on the Path to UHC. Geneva: WHO; 2016.

[53] Terwindt F, Rajan D, Soucat A. Priority-setting for national health policies, strategies and plans. In: Strategizing National Health in the 21st Century: A Handbook. Geneva: World Health Organization; 2016.

[54] International Decision Support Initiative (iDSI). The HTA Toolkit, 2018. http://www.idsihealth.org/HTATOOLKIT/.

[55] Castro H, Suharlim C, Kumar R. Moving LMICs Toward Self-Reliance: A Roadmap for Systematic Priority Setting for Resource Allocation. Management Sciences for Health; 2020.

